# Perspectives on Technology Use in the Context of Caregiving for Persons With Dementia: Qualitative Interview Study

**DOI:** 10.2196/63041

**Published:** 2024-12-13

**Authors:** Karl S Grewal, Rory Gowda-Sookochoff, Shelley Peacock, Allison Cammer, Lachlan A McWilliams, Raymond J Spiteri, Kristen R Haase, Mary Harrison, Lorraine Holtslander, Rhoda MacRae, Joanne Michael, Shoshana Green, Megan E O'Connell

**Affiliations:** 1 Department of Psychology and Health Studies College of Arts and Science University of Saskatchewan Sasktoon, SK Canada; 2 College of Nursing University of Saskatchewan Sasktoon, SK Canada; 3 College of Pharmacy and Nutrition University of Saskatchewan Sasktoon, SK Canada; 4 Department of Computer Science University of Saskatchewan Sasktoon, SK Canada; 5 School of Nursing University of British Columbia Vancouver, BC Canada; 6 School of Health and Life Sciences University of the West of Scotland Lanarkshire, Scotland United Kingdom; 7 Programs and Services Alzheimer Society of Saskatchewan Regina, SK Canada

**Keywords:** care partner, caregiving, dementia, technology, content analysis, mobile phone, technology adoption, assistive technology, support

## Abstract

**Background:**

Examining ways to support persons with dementia and their caregivers to help minimize the disease’s impact on individuals, families, and society is critical. One emerging avenue for support is technology (eg, smartphones and smart homes).

**Objective:**

Given the increasing presence of technology in caregiving, it is pertinent to appreciate whether and how technology can be most useful to a care partner’s everyday life. This study aims to further understand care partner technology use, attitudes, and the potential role of off-the-shelf technologies (eg, smartphones and smart homes) in supporting caregiving from the perspective of care partners for persons with dementia.

**Methods:**

We conducted a telephone cross-sectional survey using random digit dialing with 67 self-identified care partners of persons with dementia across one Canadian province. Participants were asked about attitudes toward technology, barriers to and facilitators for technology use, technology use with caregiving, and demographic information. Eight open-ended questions were analyzed using content analysis; 2 closed-ended questions about comfort with and helpfulness of technology (rated on a scale of 1 to 10) were analyzed with frequencies. From these data, an in-depth semistructured interview was created, and 10 (15%) randomly sampled care partners from the initial collection of 67 care partners were interviewed approximately 1 year later, with responses analyzed using content analysis.

**Results:**

Frequency analysis rated on a scale of 1 to 10 suggested that care partners were comfortable with technology (wearable technology mean 7.94, SD 2.02; smart home technology mean 6.94, SD 2.09), although they rated the helpfulness of technology less strongly (mean 5.02, SD 2.85). Qualitatively, care partners described using technology for functional tasks and some caregiving. Barriers to technology use included cost, lack of knowledge, security or privacy concerns, and undesirable features of technology. Facilitators included access to support and the presence of desirable features. Some care partners described merging technology with caregiving and reported subsequent benefits. Others stated that technology could not be adopted for caregiving due to the degree of impairment, fear of negative consequences for the person living with dementia, or due to incongruity with the caregiving philosophy. Furthermore, care partners noted that their technology use either increased or was unchanged as they moved through the COVID-19 pandemic.

**Conclusions:**

The 2 analyses were conducted separately, but there was notable overlap in the data, suggesting temporal stability of identified content. Both analyses suggested care partners’ relative comfort with technology and its use, but other care partners noted concerns about integrating technology and caregiving. Care partners’ reports of increased technology use throughout the COVID-19 pandemic may also suggest that the pandemic impacted their perceptions of the usefulness of technology, being influenced by the requirements of their reality. Future investigations should examine how to support care partners in adopting relevant technology.

## Introduction

### Background

Aging populations worldwide have created an increased prevalence of age-related syndromes, such as dementia [1]. A total of >50 million people worldwide are currently living with dementia, and 152 million are expected by 2050 [2]. Costs related to dementia were estimated to be US $263 billion in 2019 [3]. It is critical to examine ways to support people with dementia and their care partners to help minimize dementia’s impact; this may be accomplished using technology [4,5]. In this study, we explored care partners’ perspectives on whether technology can be integrated into caring for someone with dementia.

As dementia progresses, care partners have ever-increasing responsibility [6]. Their loved one’s ability to be autonomous declines with time, and the need for care increases [7]. Caregiving tasks include providing transportation, performing household work, scheduling and coordinating appointments, managing finances, providing personal care [8], and managing challenging behaviors [9]. Irrespective of whether care is informal (eg, family members) or formally paid, caring for persons with dementia is often challenging [10]. Care partners’ physical, psychological, and functional health and emotional well-being may be impacted [10-12], contributing to care partner burnout and worsened outcomes for the person with dementia [13].

A variety of services and programs support care partners of people living with dementia [14-17]. For instance, psychoeducation facilitates knowledge of the disease, skills training, and strengthening of mental and physical health [18,19]. Other services include social support [20], therapeutic intervention [21,22], and respite care [23]. Care partners benefit from these supports, reporting an increased sense of mastery and quality of life, along with reductions in perceived burden and depressive symptoms [16,18]. Care partner supports are frequently multicomponent, reflecting the many parts of the caregiving experience [22]. Interventions with multiple components confer benefits to care partners (eg, improved well-being and coping [24]), at times offering more benefit than those targeting a single area of function [25] or using a single approach [26].

One additional component to support care partners is the integration of technology to assist in caregiving activities [27-30]. Recent work has illustrated technology’s place in caregiving, facilitating better interactions [31,32] and supporting social networks [33]. Others have reported that technology has been useful in creating 24/7 backups for taxing caregiving activities or relieving caregiver anxiety [34]. Furthermore, a review of touchscreen technology interventions for care partners and people living with dementia found that all the programs positively impacted the mood and mental health of both members of the dyad [35]. Some programs included features that provided some assistance with day-to-day care (eg, medication reminders). Smartphones have also been used to assist in caregiving [35,36], including seeking information and contacting health professionals [37]. This evidence suggests that technology-driven interventions are becoming increasingly feasible and acceptable in the modern age of caregiving [27]. As these avenues are pursued, recommendations advise explicit attention to care partner needs, such as flexible administration and individual tailoring [38].

Integration of technology into the lives of people with dementia is beneficial [39-42], and recent evidence reaffirms that people with dementia can learn technologies [43]. Computer systems provide assistance in memory, deepen care partners’ insight into the person being cared for [44], and can result in increased empathy and understanding among care partners [45]. In a survey of care partners, smartphones and computers were most likely to be useful in assisting with the activities of daily living of persons with dementia [37]. Care partners reported comfort from knowing that their loved one with dementia could contact them via technology should the need arise [30].

Technologically driven interventions could address the mental health sequelae of caregiving [46-48]. For example, telehealth interventions (including telephone and blended computer-telephone modalities) for care partners of people with dementia can decrease depressive symptoms and risk of mental health problems [49] but did not appear to address anxiety, quality of life, and care partner burden [49], suggesting that interventions aimed solely at the care partner may not address the source of the mental health sequelae because the person with dementia is a part of their network [50].

When using technologies in the context of caregiving and dementia, barriers to success have been detailed [51-53] and conceptualized in models of technology adoption, such as the Technology Acceptance Model (TAM) [54,55]. For this study, we focused on off-the-shelf technology for 2 reasons: the ongoing COVID-19 pandemic and the preferences of people living with dementia to age in place [56], which can be aided by technology [57]. For many, the barriers to engaging with technology are financial [52,58]. Individuals with low socioeconomic status or belonging to an ethnic minority group are less likely to access technologies [59,60]. Lack of exposure then precipitates a double-digital divide [61]. Specifically, lack of technology exposure manifests an additional psychological barrier (eg, low confidence [62-64]) to the adoption of new technology [54,65]. Additional research suggests that gender may be related to barriers to technology use for caregiving [37,66]. For example, in a survey of informal care partners, men, on average, held more positive attitudes toward technology (eg, being willing to spend more money), while women tended to have more knowledge of the caregiving capabilities of technology [66].

In a recent study of training older adults to use videoconferencing, O’Connell et al [64] noted several additional barriers, including fear of technology and feelings of low perceived usefulness of the technology. This is consistent with related literature where people living with dementia expressed ambivalence about the use of technology [52,67,68]. By contrast, care partners have been noted to respond positively [52], even if they are unfamiliar with the technology [66]. As a final note, some participants in the study by O’Connell et al [64] expressed security concerns when using technology, such as invasion of videoconferencing rooms or “Zoom-bombing.” Privacy concerns such as these may exacerbate the barriers elaborated above and have been included in discussions of the ethics of technology in dementia care [69]. Care partners’ perspectives on the use of technology are understudied, and this knowledge gap must be given attention if we are to successfully create and modify interventions with caregiving dyads.

### Objectives

Given the increasing presence of technology in the world of caregiving, it is pertinent to ascertain whether and how technology can be most useful to a care partner’s everyday life. Broadly, this study sought to further understand care partner technology use, attitudes, and the potential role of off-the-shelf technologies (eg, smartphones and smart homes) in supporting caregiving. We explored this through the voices of current care partners of people living with dementia in the province of Saskatchewan first by way of a structured survey administered to a population-based sample of care partners. The purpose of the initial collection was to ascertain broad categories, such as barriers and facilitators to technology use, technology use in caregiving, and areas of perceived need. From this initial collection, a follow-up in-depth interview was developed and delivered via remote methods to a subset of the initial sample to gain a further understanding of technology and caregiving at an individual level.

## Methods

### Ethical Considerations

Ethics approval for this study was granted by the University of Saskatchewan Research Ethics Board. Consent for study participation was obtained verbally from the participants before both the telephone survey and in-depth interview. No compensation was offered to participants. The data presented in this paper have been deidentified.

### Recruitment

We conducted a population-based, cross-sectional survey with care partners of people living with dementia between October and December 2021 using a random digit dialing approach to call landlines and mobile phones within the province of Saskatchewan. Calls were conducted via telephone using the Canadian Hub for Applied and Social Research. We asked whether anyone in the household identified as an informal care partner for someone with dementia. We then asked to speak with the care partner and provided study information. Upon obtaining consent to participate from the person who identified as a care partner, all individuals were asked to confirm that they were care partners of people living with dementia (whether formal or informal) residing within the province before proceeding to survey administration. Sociodemographic questions involved the following topics: sex, gender, care partner age, age of the person with dementia, age dementia was diagnosed, and dementia etiology. In addition, questions were asked pertaining to the nature of the care partner relationship (eg, spouse or friend), as well as the caregiving context (eg, living together vs not and a person living with dementia living in a care facility vs living in the community). As this was a data collection effort for a larger study, not all extant variables were analyzed in this study. Then, a series of questions were asked about technology use for caregiving, including attitudes toward technology, barriers to uptake, and facilitators to using technology for caregiving. The full survey is provided in Multimedia Appendix 1. Sample characteristics for the telephone-delivered survey are provided in Table 1 (N=67).

Participants were asked whether they were willing to be contacted again for future research; 10 (15%) care partners were randomly selected from this subsample of 67 care partners for a second in-depth, semistructured interview. We had hoped to sample purposefully to capture diversity within the interviews, but the selection was random because there was no way to link survey responses to identifying information in accordance with institutional ethics guidelines. This in-depth interview was conducted approximately 1 year (August 2022) after the initial telephone survey. The guiding questions for this interview were informed by the literature and collective responses of the telephone survey (ie, categories in the data). A team of clinician researchers (KSG, MEO, AC, and SP) helped to develop the semistructured interview guide, which involved a collection of iterative discussions and multiple drafts. Sample characteristics for the telephone-delivered survey are provided in Table 2, and the semistructured interview guide is provided in Multimedia Appendix 2. Interviews were conducted remotely by KSG through videoconferencing, recorded, and subsequently transcribed verbatim.

**Table 1:** Sample characteristics for care partners who completed the telephone-delivered survey (N=67).

Characteristics		Values
**Sex, n (%)**		
	Male	17 (25)
	Female	50 (75)
**Age of care partner (y), mean (SD)**		
	Male	72.00 (16.56)
	Female	64.34 (12.15)
Age of person with dementia (y), mean (SD; range)		82.87 (7.30; 59-97)
**Person to whom care partner was providing care, n (%)**		
	Parent	27 (40)
	Spouse	27 (40)
	Sibling	2 (3)
	Grandparent	1 (2)
	Other relationship (eg, uncle or aunt, cousin, and partner)	10 (15)
**Care partner’s impression of the cause of the dementia reported by care partner, n (%)**		
	Alzheimer disease	11 (16)
	Vascular dementia	4 (6)
	Dementia due to Lewy bodies	2 (3)
	Medication induced	2 (3)
	High blood pressure or second hand smoke	1 (2)
	COVID-19 or concussion	1 (2)
	Brain fistula	1 (2)
	Frontal lobe dementia	1 (2)
	Diabetes	1 (2)
	Stroke	1 (2)
	Parkinson disease	1 (2)
	Not reported	40 (60)

**Table 2:** Sample characteristics for the care partners who completed in-depth interviews (n=10).

Characteristics		Values
**Sex, n (%)**		
	Male	2 (20)
	Female	8 (80)
Care partner age^a^ (y), mean (SD; range)		67.4 (9.45; 49-78)
Duration in care partner role^a^ (y), mean (SD; range)		3.52 (2.67; 14 mo-10 y)
**Person to whom care partner was providing care, n (%)**		
	Spouse	5 (50)
	Parent	4 (40)
	In laws	1 (10)

^a^Care partner age and duration in the care partner role were not significantly different for male and female individuals, so only 1 mean is reported.

### Analysis

Descriptive analyses of quantitative and survey data were performed using SPSS software (version 27.0; IBM Corp). Descriptive statistics, including means and frequencies, were used to describe the study sample. Limited univariate analysis was completed with scale-based questions (ie, ratings from 1 to 10), using 2-tailed *t* tests to explore self-reported ratings of comfort with and helpfulness of technology for caregiving.

### Content Analyses

Two separate content analyses [70,71] were conducted: one for the open-ended questions from the telephone-delivered survey and another for the in-depth interviews. The analyses were directed [72] using the TAM [53,54] as a conceptual framework. The TAM has frequent support [63,73,74], suggesting that perceived usefulness and perceived ease of use are beliefs that influence attitudes toward and use of a given technology [53]. The TAM has been expanded and differentially applied [75], with recent application to rural and older adult contexts [61,63]. In these expanded models, external variables (ie, age, education, income, and social and cultural background) act through perceived usefulness, access barriers, and perceived ease of use to impact attitudes toward and use of technology [61]. In addition, these analyses were conducted using pragmatism [76] as a paradigmatic frame. Pragmatism advocates for the method that is most appropriate for a given research problem [77] to keep focus on the consequences of research [78]. In this study, we wanted to explore what would matter most to care partners at the intersection of technology and caregiving [76,79].

For the telephone-delivered survey, KSG first read and reread responses to each open-ended question to support familiarization with the data [70]. KSG created a preliminary codebook based on this familiarization. The codebook was then given to another investigator (RG-S), who coded the first 10 to 15 responses for each question as a pilot test of the codebook. The codebook was refined by KSG and RG-S following this pilot phase; memos were used to document any new codes or ideas about the data. Next, the coding of all responses was completed independently by 2 coders (KSG and RG-S), who subsequently met to resolve discrepancies. Another investigator (MEO) was available to resolve the remaining coding issues. Once coding was completed, KSG and RG-S identified initial categories. Subsequently, KSG, RG-S, and MEO collaboratively discussed and refined these categories for each question. The in-depth interviews were analyzed using a similar multistep content analysis. KSG began with data familiarization and created a pilot codebook, which was refined through reflexive memoing and discussions with MEO, AC, and SP. KSG was the sole coder, although MEO, AC, and KSG engaged in collaborative discussions surrounding processes and categories.

**Figure 1:**
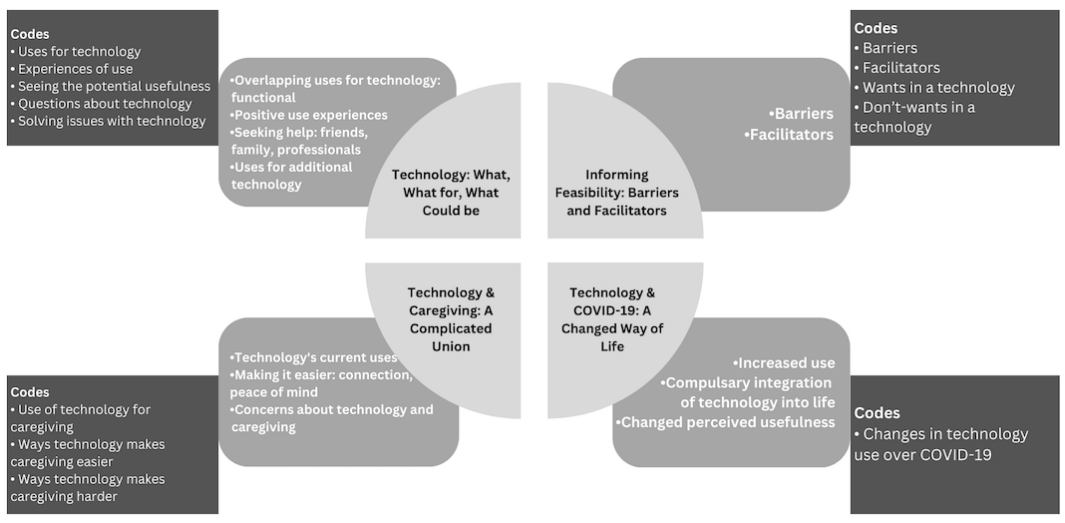
Map of data aggregation for the 10 in-depth care partner interviews. Codes are presented on the outside of the image, flowing inward to data organization, and finally to labeling the categories. Codes, processes, and categories were all directed by our conceptual framework, the Technology Acceptance Model.

Several methods of rigor were used in both content analyses. First, to support confirmability, a comprehensive audit trail was kept to document notes about the context of the research, methodological decisions (eg, codebook revisions), and the analysis process [80]. Data mapping [70] was used in the analysis of the follow-up interviews to track the evolution of categories due to the increased abstraction from codes to categories (Figure 1). To ensure credibility, each coder used reflexive memoing to document interesting findings, similarities, differences, emerging patterns, and relationships throughout the coding process [80]. Third, our research team consisted of multidisciplinary researchers (eg, psychology, community health and epidemiology, and nursing) with diverse skills and theoretical perspectives, which provided a more insightful and nuanced approach to interpreting our study’s findings. Finally, we used low inference methods, allowing the findings to remain close to the experiences and ideas of participants, lending further support to confirmability [80].

## Results

### Overview

Analysis of care partner responses to several questions about their use of and attitudes toward technology using a 10-point scale are provided in Table 3.

**Table 3:** Results of frequency analyses of quantitative data from care partners who completed the telephone survey.

Survey question		Average rating, mean (SD)
**Comfort with technology^a^ (1=not comfortable at all and 10=very comfortable)**		
	Wearable technology (n=39)	7.94 (2.02)
	Smart home technology (n=18)	6.94 (2.09)
Helpfulness of technology for caregiving^a^ (n*=*62; 1=not helpful at all and 10=very helpful)		5.02 (2.85)

^a^No difference between male and female participants.

### Telephone Survey

Content analysis of the telephone survey is presented in this section, first with descriptions of reported uses, parsed by wearable and smart home technologies. Subsequently, categories pertaining to experiences of use, understanding technology use in daily life, fears about technology for caregiving, and technology use during the COVID-19 pandemic are discussed for both technologies together.

#### Reported Uses

##### Wearable Technology

Of the sample of 67 care partners, 41 (62%) reported using wearable technology in the past (eg, an Apple Watch, an iPod in a carrier, or a smartphone). Overwhelmingly, wearable technology was reportedly used in a functional manner. Uses included fitness tracking; entertainment (eg, music and news); communication (eg, email, texting, and FaceTime [Apple Inc]); shopping; finances; looking up information; and keeping track of appointments. The following quotes reflect the variety of functional uses reported:

Pretty much everything, google search, email, telephone calls, anything I can think of, research.

Surfing the web and playing games entertainment and news uhm just different things like that texting with family keeping in touch with family.

Communicating, looking things up, recording my phone numbers, appointment dates.

A small number of individuals explicitly reported using wearable technology for caregiving activities. One person indicated that they used wearable technology to store “medical information like her [care recipient’s] immunization and [care recipient’s] ID” to make outings more efficient. Other care partners reported using their smartphones to share memories with their loved one with dementia, including “pictures on the phone of his family, his children, his grandchildren.” Finally, other care partners reported using their wearable technology to help keep contact with their care recipient either in a monitoring capacity (eg, “I just need to make sure where he’s at all times”) or for communication (eg, “with caregiving just the Facetiming that’s all I really used it for”).

##### Smart Home Technology

From the sample of 67 care partners, 17 (25%) reported using smart home technology in the past (eg, Alexa [Amazon.com], Google Home [Google LLC], or iHome [SDI Technologies]). Predominantly, care partner’s use of smart home technology fell under the category of assistance with day-to-day functional tasks. Tasks included communication, looking up information (eg, weather and song names), entertainment (eg, music and news), setting alarms, and shopping. Furthermore, a small group of care partners described using their smart homes for remote monitoring (eg, a camera on the front door, changing temperature or lighting, or home security). The following quotes exemplify the functional and monitoring uses reported by care partners:

We use it for music, news, weather, keep track of shipments, some security, hook up to fire smoke detectors and water and lots of things.

Alexa for alarms and stuff like that and listening to radios and we do have a smart doorbell so when we’re not home we can see who comes to the door.

#### Experiences of Use

When care partners described their experiences with wearable and smart home technology, 3 types of experience were found. Mostly, care partners spoke positively about their experiences, using descriptors such as “seamless,” “easy,” or “helpful.” One care partner noted the following:

I love it. I wouldn’t change it. I would upgrade, get something in the bedroom or downstairs.

A second group endorsed a neutral stance on their experiences. These care partners used descriptors such as “fine,” and responses suggested that although these care partners were not as enthused as those with positive experiences, the technology was usable and, to some degree, acceptable:

It was ok I wouldn’t say I was thrilled with it I would say neutral.

The third group of care partners described hesitancy in engaging with technology. Responses indicated that these individuals would engage with technology if they needed to, but issues such as tedium, frustration, and intimidation presented barriers for them:

I’m not big on techie stuff. I don’t care for it, that’s why I only use it when I have to, [when I] really need help I call the kids and they help me... I had a computer before but now I have this iPad because my son gave it to me, said that I needed it, it’s simpler he said. I’m really not good with computers and I don’t have any patience with them either.

Woven within experiences of smart home use were issues with the smart home technology. Issues frequently described included a problem experienced with setup, maintenance, or the use of smart home technology (eg, “I found it fine, but hard for husband to use it since he has an accent.”). One care partner reported a negative experience with their smart home, citing concerns with its invasiveness and its necessity in their lives:

I did not like it was on all the time and listening to what I was saying. I did not find it necessary either.

#### Understanding Technology Use in the Context of Daily Life

Overwhelmingly, care partner’s responses reflected how technology enhanced the performance of their day-to-day activities. Repeatedly articulated were the notions of ease, speed, and increases in the care partner’s access to information (eg, “I mean it’s awfully convenient, it’s fast”). Relatedly, responses reflected an appreciation of technology’s utility in helping with functional tasks. Examples included assistance with appointments, communication, and the multipurpose nature of technologies (eg, a participant described their smartphone “doubl[ing] as a camera, flashlight, and measuring device”). One care partner described preferring the ability to be available if their mother needed assistance, finding “security” in the technology.

Care partners reported various dislikes about technology that can be best conceptualized as barriers to use in their daily lives. First among these was a lack of technological knowledge. Care partners described that they found it “hard to keep up with everything” and “hard to learn it all” because it “keeps changing*.*” Related to a lack of knowledge were reports of issues with technology simply not working as required and unsuccessful attempts at troubleshooting. Furthermore, care partners expressed concerns regarding the privacy and security of the technology. Concerns included “invasiveness,” security threats (eg, phishing scams), and the tracking and storing of information. Several care partners also mentioned cost as a barrier, referencing both the price of equipment and subscription services. Finally, several care partners endorsed a dislike for how technology changed the world around them. Some found technology “overpowering” and “distracting,” while others disliked the feeling of constant connectivity and availability, as well as the changes to communication styles (eg, “people are so dependent on that they can’t think for themselves”).

#### Fears About Using Technology for Caregiving: Feasibility and Adverse Impacts

When asked to describe their fears about using technology for caregiving, 2 categories emerged. First, care partners expressed concerns regarding the feasibility of technology for caregiving. Reported feasibility concerns often centered around the person with dementia being unable to learn the technology (eg, “well my concern is that caregiving for elderly people the elderly person isn’t often able to learn or utilize the technology I guess”) or perceptions that the addition of technology would not be helpful or applicable. As an example, one care partner noted that “because caregiving is a hands-on project,” technology may not be an appropriate addition, with another relating as follows:

You can see them, but you don’t know how they are physically or what they look like or what’s happening without actually being there.

Care partners also expressed fear of the negative impact of technology on the person living with dementia or the care partner. One fear was about security, namely, being vulnerable to phishing scams due to a lack of technological understanding. Furthermore, care partners stated that the addition of technology may have adverse psychological consequences for their loved one with dementia, including increased anxiety and paranoia. One care partner shared their perspective as follows:

... because people with dementia don’t understand the electronic technology like televisions and things like that, so it can increase their anxiety and stress and confusion rather than decrease it. Like Facetiming with someone with dementia doesn’t usually work very well and it can also create just more anxiety for them.

#### Technology Use and the COVID-19 Pandemic

When asked about technology use during the COVID-19 pandemic, 64% (43/67) of the care partners indicated that they had increased their use. When asked why, responses were consistent with a category of increased perceived usefulness for functional activities. Notable examples included communicating with people while physical visiting was restricted (eg, “I was not able to visit my wife, so I made arrangements for FaceTime visits”); attending appointments and leisure activities (eg, “doctor appointments became telephones consults, exercise programs on zoom*...*”); attending work; and completing household tasks (eg, “I mean in my life time I have never paid a bill on a computer and you know now I do banking on computer”).

The remaining care partners (24/67, 36%) described that their use of technology had not changed because of the COVID-19 pandemic. When elaborating, a small number of respondents indicated that technology had never been critical in their lives, with some being from a farming background or citing themselves as preferring outdoor activities, such as gardening, that the COVID-19 pandemic did not impact. The remainder of care partners indicated that they were using technology to a relatively high degree before the pandemic (eg, “No. Well I haven’t noticed any difference since COVID or before*.*”).

### In-Depth Interviews

Demographics for interviewees are provided in Table 2. Our analysis of this collection of interviews yielded the following categories.

#### Technology: What, What for, and What Could Be

Care partners were asked at the beginning of each interview to describe their experiences with technology in daily life to gauge what came to mind spontaneously. Technology listed included computers, email, cell phones, televisions, videoconferencing programs (eg, Zoom [Zoom Video Communications]), monitoring technology (eg, Lifeline medical alert system), portable technologies (eg, e-reader and iPad), and faxing. Uses included communication, assisting in daily life (eg, appointments), monitoring the person with dementia, and keeping apprised of care plan changes of the person with dementia. Care partners were then directly asked whether they used a smartphone, smartwatch, or smart home technology. All 10 care partners reported using a smartphone: 7 (70%) used iPhones and 3 (30%) used Android devices. Of the 10 care partners, 3 (30%) reported using smartwatches, with 2 (20%) others endorsing past use. Three care partners described using smart home technologies, including the Nest system (Google LLC), Ring Doorbells, Google Home, and Amazon Alexa.

Care partners described using these devices overwhelmingly for functional purposes, such as communication, obtaining information, entertainment, and health care needs (eg, vaccine information and heart rate monitoring). In addition, experiences of using smartphones, smartwatches, and smart home systems were largely positive within this subsample. Furthermore, when care partners encountered problems with or had questions about their technology, they described consulting friends and family (eg, kids and grandkids “are way better at it than I am”), independently seeking solutions (eg, Google or YouTube [Google LLC]), or receiving professional assistance.

Some care partners also discussed the potential usefulness of technologies (ie, smart homes or smart watches) they were not currently using. Although frequently unsure of what they might use it for, care partners described these technologies as intriguing conveniences (eg, “It’s a bit of a novelty”). One care partner expanded, noting their interest in “any technological applications that would help me live independently as long as possible*.*” Furthermore, a care partner who self-identified as a visible cultural minority individual (more detail is not provided to maintain confidentiality) related the potential of wearable GPS technology to address elopement concerns. They noted that they would like to monitor their person with dementia and locate them by leveraging their community rather than involving authorities “cause there’s a trust issue with the police, right?” Moreover, 1 care partner indicated no use in their lives for the additional technology due to functional overlap with currently owned technology (eg, smartphone).

#### Informing Feasibility: Barriers and Facilitators

##### Barriers

Care partners described a series of barriers relevant to their engagement with technology. These included concerns mentioned in the telephone survey, such as security and privacy (eg, “they can get talked into something so easily because people are so good at duping them”) and a lack of knowledge. Lack of knowledge was a nuanced barrier, including descriptions of not knowing where to start with technology (eg, “I don’t know what I don’t know”), being unsure of what technology could be useful to them (eg, “I don’t know what the technology is out there that would be helpful for me”), or that their “plate was too full” to learn something new. Relatedly, some care partners felt too intimidated to learn about technology (eg, “...its so intimidating and frustrating and I would be afraid to mess it up.”). Interestingly, although cost was identified as a barrier, most interviewees noted that it was not a chief concern, especially if they understood the tangible benefits of adding technology to their life:

I would have to weigh the cost benefit analysis... if there was a technology that would be helpful, I have the means to provide that.

In addition, care partners described aspects of technology that might deter them from use. These included features such as unnecessary invasive passive monitoring, small text or picture size, and too many notifications. Notably, care partners sometimes had difficulty identifying features they would avoid due to a lack of knowledge.

##### Facilitators

Care partners also described a variety of factors that would facilitate engagement with technology. The most prevalent facilitator was the presence of support. Whether it was supportive teaching or support for setup and troubleshooting, care partners felt support made using technology easier. The following examples detail the value of available support:

It was really comforting for any of us to know you just had to click here and somebody would come and say “I’ll take care of this for you.”

When I couldn’t make my mom understand how to use the remote to connect to her TV and get it working, To have a care aide that would just come in and know what buttons to push meant so much... its just such a relief to me. It just takes the stress away from me.

In addition, care partners emphasized several aspects of technology that would facilitate use. These included simplicity and ease of use (eg, big screens, easy charging, and limited buttons); failsafe mechanisms in case of internet outages; ability to virtually drop in and check on a loved one; games to facilitate cognitive engagement; ability to set reminders; ability to program phone numbers for voice-activated calls; and any features that would facilitate interaction between care partner and care recipient.

#### Technology and Caregiving: A Complicated Union

Care partners reported using technology in their caregiving in varying ways. These included virtual visits and appointments; looking up dementia-related information; attending virtual support groups; providing comfort to their loved one (eg, “a little bit for providing music... it’s a very calming thing if he’s feeling anxious or agitated”); reminiscence (eg, “he looks up things that we have on this Facebook page... its about old-time days. And he’s back in old time days now with his dementia so he really enjoys looking up that information”); and recording memorable moments, such as “her playing guitar*.*” Furthermore, sharing pictures via cell phone was used to facilitate engagement in daily life:

...because he’s not as mobile as he used to be, he can’t get out into the garden and see all the new blooms and stuff. So I take tons of pictures of the yard.

Care partners also related that technology made caregiving easier for them in some ways. Technology appeared to facilitate social connection with their loved ones, as during visits, “*...*there isn’t a whole ton of things to talk about all the time so it’s nice to look at things on the phone, pictures and stuff*.*” Others described technology as “a safety thing*,*” providing peace of mind:

We went for haircuts and I forgot my phone, and I just felt very disconnected, I felt a little bit anxious because I thought if we had an emergency I have no way of connecting to anybody...

However, care partners also expressed reservations about using technology for caregiving. For instance, concern was raised about whether the person with dementia would benefit from the technology or whether it would be useful for their caregiving. This took 2 forms. First, there was concern about a lack of interest in technology:

Like I said [person with dementia] doesn’t have internet... I’m sure he would not want to add technology. Otherwise, he would have had it himself.

Second, care partners reported worrying that the person with dementia was too impaired to meaningfully interact with technology, as illustrated in the following quotes:

For [person with dementia] she would reach out because she would see me on the iPad a few feet in front of her she’d reach out and knock the iPad over and it’s looking up at the ceiling and there’s nobody around to help so that’s the end of that.

With dementia, I’m not sure that my mother in law would “get” it, or that she would find a use for it.

In addition, care partners cited a hesitancy to merge technology with their caregiving for fear of upsetting their loved ones:

No, because my mom might freak. Sometimes shes good—we’ve never done FaceTime with her... I call her on the phone almost every day just to check on her. That’s how we used to always communicate. If we take our phones with us to show her pictures, she’s interested, but we’re not sure how she would react to FaceTiming... She actually ordered an iPad but once they put her on meds she got terrified everybody’s listening in.

That’s where [person with dementia] is right now, where if something doesn’t work it frustrates them and they want nothing to do with the technology.

Finally, some care partners saw technology and caregiving as a potential mismatch, viewing caregiving as something that was hands-on. One care partner detailed a powerful example, fearing missing vital information if caregiving was too reliant on technological connection:

I wouldn’t have known that she lost 30 pounds in over a month, 5 weeks. With clothes on, if you did with FaceTime, you’d just see the face, you wouldn’t see the body to know stuff was going on and for other people you never know if someone’s getting abused cause all you ever see is the face.

#### Technology and the COVID-19 Pandemic: A Changed Way of Life

Most (9/10, 90%) care partners reported an increase in technology use through the COVID-19 pandemic for entertainment (eg, video streaming) and medical appointments consistent with the telephone survey nearly a year before. Moreover, care partners detailed that technology was integrated into their lives by necessity:

They put you in a situation where you kinda didn’t have a choice.

The COVID-19 pandemic forced families and support networks apart; many care partners emphasized the importance of using technology to maintain connection:

You had to use that because otherwise you don’t see your family.

Even though their memory may be short, they need social contact and I’d phone her three, four times a day just to see how she was doing so the phone was so important.

Interestingly, the forced integration of technology into daily life appeared to change perceptions of usefulness among some care partners. It seemed that the shift in global circumstances allowed for a re-evaluation of the way things were done in daily life and how technology could be beneficial:

There were certainly applications I had never even heard of before that became a part of my life... it became a substitute for personal meetings and I think I learned the benefits from it. Those things aren’t going to go away.

Just the exercise program from the Alzheimer’s Society that I was mentioning to you, [program name], was only offered in [urban center] and [urban center] prior to the pandemic, then they started offering it virtually. Now there are people from [rural communities]. Those people would never be able to access that service, and now they can so hopefully it will continue because then people in rural Saskatchewan can be better serviced through many things that are already in existence, but they just couldn’t get to a big city to do that.

## Discussion

### Principal Findings

Overall, participating care partners were relatively comfortable with technology, using it overwhelmingly for functional purposes and to assist with caregiving. Although experiences using technology varied, care partners enjoyed the convenience of technology in enhancing daily living. Several barriers to technology use were described, including cost, security and privacy concerns, lack of knowledge, and undesirable features of the technology. Conversely, facilitators included the presence of technology support (ie, a support person), enhancing the performance of daily activities, and features promoting ease of use. Care partners also reported using technology in their caregiving in a variety of ways, with some expressly noting how it facilitated connection and safety. However, concerns about the integration of technology and caregiving were also expressed, including lack of feasibility due to their loved one’s impairment, fear of negatively impacting the person with dementia, and feeling technology would be neither helpful nor applicable. Furthermore, care partners reported an increase in technology use in their daily lives throughout the COVID-19 pandemic, with the pandemic changing perceptions of technology’s usefulness.

The results of each content analysis were reported separately to reflect the fact that substantial time had lapsed between data collections. Although there was unique richness to each analysis (eg, experiences of use and elaboration on facilitators, such as support), there was notable overlap in the data. Both analyses suggested care partners’ relative comfort with technology, using it functionally and for caregiving. There was also overlap in barriers, including cost, security and privacy concerns, and lack of knowledge. Moreover, concerns about the integration of technology and caregiving were replicated, including lack of feasibility, worries of negative consequences, and feeling that technology would not mesh with their caregiving. Care partner’s reports of increased technology use throughout the COVID-19 pandemic were consistent across analyses. Given that there was almost a year between data collection waves, this substantial overlap could suggest temporal stability in addition to the uniqueness of each content analysis.

### Comparison With Prior Work

Care partners in our sample reported using technology overwhelmingly for a variety of functional purposes (eg, communication, information and entertainment, and appointments), with some describing the use explicitly for caregiving. These findings align with prior reports of care partner technology use [81]. Examples include care partner use of smartphones and computers for maintaining social connections and contacting health care professionals [37], as well as in caregiving tasks, such as personal care and leisure [82]. Furthermore, our sample was relatively comfortable with technology and described their experiences of use as largely positive, with some experiences being neutral or negative. Interestingly, positive previous experiences with technology have been reported as a key factor in technology adoption for older adults [63]. By virtue of exposure, increasing care partner’s technology use in their daily lives will likely increase positive experiences, leading to fewer psychological barriers to engagement [61,65].

Barriers reported by care partners included cost, security and privacy concerns, and undesirable features. Similar barriers to technology use in the context of dementia have been described in a recent systematic review, including cost; ethical issues; and issues with features, such as poor sound quality and small font sizes [52]. However, our analysis suggested that the cost can be recontextualized as worthwhile if care partners understood the benefit of the technology weighed against its cost, underscoring the importance of perceived usefulness [54,63].

Another key barrier was a lack of knowledge. Some care partners did not know where to start with technology or were unsure of which technology could be useful. Others felt too busy to learn anything additional, perhaps because the learning process or their involvement would be too burdensome [82,83]. Interestingly, another study reported a lack of knowledge interfering with care partners’ adoption of new technology (ie, lack of understanding or uncertainty in use [84]). This lack of knowledge may lead to approach anxiety or low confidence [63,85,86]. We saw this in our data, with care partners reporting feeling intimidated and fearing getting things wrong. Returning to the TAM [53], lack of knowledge likely acted as a pervasive barrier to perceived usefulness [54] as the category ran throughout the telephone survey and all in-depth interviews. This may help explain the discrepancy between the ratings of comfort with and helpfulness of technology, such that care partners were not aware of the ways in which technology could help them.

Three major facilitators for technology use were described by care partners: the presence of support during setup and troubleshooting, enhancing performance of daily activities, and the presence of desirable features. The need to support the uptake and use of technology in the context of dementia care was also reported in a pilot study of Fitbits [87]. The authors noted the necessity for technical support throughout their intervention. Support has been described by our group as critical to technology adoption, especially for those who are hesitant [61,64]. Consequently, hearing about its importance directly from care partners is particularly powerful. Furthermore, viewing technology as an avenue to enhance daily life leaves space for care partners to see technology fitting into their routines. To this end, Mortenson et al [88] reported that care partners found technology enhanced caregiving and their daily lives. Important to this enhancement was the collaboration between providers, the person with dementia, and the care partner to maximize the tailoring of technology to the setting.

Finally, our care partners articulated several features (eg, simplicity and fostering connection) that would increase their likelihood of using technology. Extant literature supports the presence of specific features as a facilitator, with care partners preferring technology that is easily accessible, easy to use, and familiar to them [66], as well as useful in day-to-day life [35]. Interestingly, the facilitators identified in this study directly align with elements of the TAM [53,54]: perceived usefulness and perceived ease of use. Specifically, convenience, help with daily tasks, and desirable features would drive up perceived usefulness, while the presence of support would buttress perceptions of ease of use, precipitating an increased likelihood of using technology.

Our findings suggested that care partners were open to integrating technology and caregiving, with some already doing so to make their lives easier and bring peace of mind. A similar sentiment was outlined by other care partners, who used technology to help with caregiving and leave more room for respite [89]. This peace of mind could also increase the potential for autonomy of people with dementia, allowing care partners to take a break (S Green [MEd] and N Stewart [PhD], personal communication; November 1, 2021; [90]). Relatedly, we found that technology alleviated fears about the safety of people with dementia despite some concerns about invasiveness. These findings replicated existing literature on the importance of centering care recipient safety when using technology [88,91] while walking a line between providing care and being too invasive [92,93].

These noted positives were contrasted by concerns about the union of technology and caregiving. Our data suggested a hesitancy to add technology because of a fear of negatively impacting the person with dementia. To the best of our knowledge, hearing about this fear from care partners is a novel finding, which is consistent with some previous literature suggesting that new technologies could induce a negative reaction from people with dementia [94]. Another issue in the union of technology and caregiving was a lack of interest in bringing technology into caregiving. Other studies have reported people with dementia as resistant to technology in their lives [52], perhaps due to ambivalence or perceived lack of relevance [95].

Our findings also suggest that technology may not be feasibly integrated due to the impairment of the person with dementia. The degenerative nature of dementia has been suggested to complicate the timing of introducing technology [96-98]. This may indicate an optimal time when the care partner has a low enough burden to learn technology and the person with dementia is at a level of functioning where they can be an active participant [52,94]. Moreover, several care partners saw caregiving and technology as incompatible because caregiving is a hands-on job, and current technology cannot replicate these tasks. A similar concern was also recorded in a recent qualitative analysis. Xiong et al [84] reported that, for some care partners, technology may not be compatible. Care partners may rely on established caregiving routines exclusive of technology due to existing familiarity with these tasks and the ability to preserve a level of in-person interaction, which technology may erode [52,84]. Reflecting on these findings, it is possible that existing technologies fall short with respect to their feasibility across care partner contexts, but future advancements in technological designs may be more successful.

Furthermore, care partners reported distinct increases in the use of technology during the COVID-19 pandemic, especially for social connection, health care, and functional tasks. Increased use of technology through the pandemic has been a recurrent finding in several studies of older adults in Canada [99] and Europe [100]. In addition, the increased use of virtual health care in the context of dementia is not surprising, given the necessary health system shifts [101]. Specific to care partners, recent qualitative analyses have described new or increasingly frequent engagement with technology-based communication to maintain social connections [89,102,103], as well as reducing boredom through technological means (eg, streaming [89]). Thus, converging evidence suggests the importance of technology for maintaining bonds and assisting in daily life through the COVID-19 pandemic and beyond.

Our results further suggested a potential mechanism for increased technology uptake. Care partners reported changing their technology use during the COVID-19 pandemic both recreationally and functionally, with many articulating that they had no choice (eg, to communicate with family). Such forced contact could indicate that the COVID-19 pandemic changed the perceived usefulness of technology in care partners’ lives (ie, a COVID-19–influenced TAM [104]) out of necessity (eg, due to isolation [105]). To this end, Haase et al [99] reported that 55.9% of their sample adopted new technology during the pandemic, perhaps partly due to social motivations through the COVID-19 pandemic. As a part of the TAM [54], perceived usefulness is a key factor influencing attitudes and behaviors. It is possible that the pandemic acted as a catalyst, producing an environment where technology was perceived by care partners as too useful to ignore. Given this unique opportunity of heightened perceived usefulness, supporting care partners who are unsure about how to join the technological world is critical to avoid exclusion [106,107]. Potential avenues for engaging care partners could be clear instructions [99] or goal-oriented individualized teaching [104].

### Limitations

This study had several limitations. First, to keep the survey brief and the interview focused, a minimal number of demographic-related questions were asked. In addition, there was an element of response bias in that participating care partners may have been using technology at a higher frequency than the general care partner population. Relatedly, these individuals may have been more likely to view the adoption of technology more favorably by virtue of volunteering to participate. Furthermore, the random digit dialing approach introduced an element of nonresponse bias [99,108]. Because of random digit dialing, we cannot know the reasons for refusals (eg, lack of interest and not feeling that they used enough technology to contribute) and, therefore, do not have a response rate. However, the random digit dialing approach likely afforded us the opportunity to capture an understudied population (ie, rural caregivers in Canada) using the telephone rather than other recruitment means requiring preselection as these individuals may not have responded to other means of study (eg, web-based surveys).

Furthermore, many participants in the telephone survey did not comment on the cause of dementia in the person they cared for. This missing data limited the generalizability of our findings as the needs and preferences of care partners may vary by etiologies or comorbidities.

### Conclusions

Care partners described being relatively comfortable with technology, using it to help with functional tasks and in their caregiving. Experiences with technology ranged from positive to neutral and negative. Barriers to technology use were identified, including cost, lack of knowledge, security or privacy concerns, and undesirable features. Facilitators included access to support and the presence of desirable characteristics. Although some care partners were using technology for their caregiving, others were concerned that technology would not be feasibly adopted for caregiving or that doing so would have negative consequences for the person living with dementia. The COVID-19 pandemic resulted in many care partners reporting increased use, as well as a changed perception of the usefulness of technology, perhaps out of necessity. The substantial overlap between the 2 content analyses, although data were collected approximately 1 year apart, suggested the temporal stability of identified categories. Future investigations should examine how to support care partners in adopting personally relevant technology.

## Appendix

Multimedia Appendix 1 Telephone-delivered care partner survey.

Multimedia Appendix 2 In-depth semistructured interview guide.

## Abbreviations

TAM: Technology Acceptance Model

## Data Availability

The datasets generated during and analyzed as a part of this study are not publicly available due to restrictions dictated by the University of Saskatchewan Research Ethics Board surrounding the identifiable nature of the raw data of a vulnerable population.
