# Supplementary material for: Perspectives on Technology Use in the Context of Caregiving for Persons With Dementia: Qualitative Interview Study
Source: JMIR Form Res. 2024 Dec 13;8:e63041. doi: 10.2196/63041 (PMC11681293; doi:10.2196/63041)
Supplement: Multimedia Appendix 1 [file formative_v8i1e63041_app1.docx]

**Appendix 1: Telephone-Delivered Care Partner Survey**

**Care Partner Survey**

**(November 2021)**

**INTRODUCTION**

INT02.

Hello, my name is  ***(FIRST NAME ONLY)*** and I am a student calling from the University of Saskatchewan. We are conducting a ten-minute telephone survey focusing on attitudes towards technology use in caring for people with dementia.

INTRO2.

Is there anyone in the household involved in the care of someone who has dementia?

1. Yes, speaking ***CONTINUE***

2. Yes, I’ll get him/her ***REPEAT INTRODUCTION AND CONTINUE***

3. Not available ***ARRANGE CALLBACK 🡪 HIT “ESC” ON YOUR KEYBOARD –***

***REQUEST RESPONDENT FIRST NAME AND ARRANGE CALLBACK***

4. Refused to Transfer

INT03.

I would like to invite you to participate in this short survey. Participation is voluntary, and you can stop the survey at any time. You can skip any questions you don’t want to answer. This call will be recorded for quality control purposes. None of the answers that you provide will be linked back to you personally. There are no known risks to participating in this survey. If you have any questions or concerns, you may contact the researcher, Dr. Megan O’Connell, at 306-966-2496. This research project has been approved on ethical grounds by the University of Saskatchewan Behavioural Research Ethics Board. Any questions regarding your rights as a participant may be addressed to that committee through the Research Ethics Office at [ethics.office@usask.ca](mailto:ethics.office@usask.ca) or toll free at 1-888-966-2975.

Are you willing to participate?

1. Yes ***CONTINUE***
2. No ***THANK AND END INTERVIEW***
3. Later/Not right now ***ARRANGE CALLBACK*** 🡪 ***HIT “ESC” ON YOUR KEYBOARD –***

***REQUEST RESPONDENT FIRST NAME AND ARRANGE CALLBACK***

Q1A.

To confirm, you are 18 years of age or older, are living in Saskatchewan, and are *currently* involved in the care of someone who has dementia?

***(INTERVIEWER NOTE – PEOPLE WHO USED TO BE A CAREGIVER FOR SOMEONE WHO HAS DEMENTIA OR ARE FORMAL CAREGIVERS DO NOT QUALIFY – ENTER NO)***

1. Yes (to all of the above) ***CONTINUE***
2. No (to any of the above) ***THANK AND END CALL***
3. (Refused) ***THANK AND END CALL***

Q1.
Thinking about the person you are caregiving for who has dementia, what is their relationship to you?

1. Parent
2. Spouse
3. Sibling
4. Child (son/daughter)
5. Grandparent
6. Friend
7. Other extended relative (e.g., aunt, uncle, cousin) (SPECIFY)
8. Other non-relative (SPECIFY RELATIONSHIP)
9. (Refused)

Q2.
Do they live with you?

1. Yes ***CONTINUE***
2. No, they live elsewhere ***SKIP TO Q5***
3. (Refused) ***SKIP TO Q5***

Q3.
Do you provide all the care, or do you have other help?

1. Yes, I provide all the care **SKIP TO Q13**
2. No, I have help ***CONTINUE***
3. (Refused) ***SKIP TO Q13***

Q4.
Is this formal/paid help like homecare or informal help like friends and family?

1. Formal help **SKIP TO Q13**
2. Informal help **SKIP TO Q13**
3. (Refused) **SKIP TO Q13**

Q5.
Does the person you care for live in a facility, or do they live in their own apartment or home in the community?

1. In a facility ***SKIP TO Q8***
2. Own apartment/home ***CONTINUE***
3. (Refused) ***SKIP TO Q13***

Q6.
Does the person you care for live alone, or do they live with someone else?

1. Live alone
2. Live with someone else
3. (Refused)

Q7.
Does the person you care for have formal help like homecare or is all the caregiving provided by friends and family?

1. Formal help ***SKIP TO Q10***
2. Informal help ***SKIP TO Q10***
3. (Don’t know) ***SKIP TO Q10***
4. (Refused) ***SKIP TO Q10***

Q8.
Does the person you care for live in a long-term care or assisted living facility?

1. Yes ***SKIP TO Q10***
2. No ***CONTINUE***
3. (Refused) ***SKIP TO Q10***

Q9.
What kind of supports are provided in the facility they live in?

1. (RECORD RESPONSE VERBATIM)
2. (Don’t Know)
3. (Refused)

Q10.
What role do you play in caregiving?

1. (RECORD RESPONSE VERBATIM)
2. (Refused)

Q11.
How often are you in contact with the person you care for? Is it…?

**(READ LIST)**

1. Daily
2. Weekly
3. Monthly
4. Less than once a month
5. (Refused)

Q12.
How do you usually have contact with the person you care for?

***(SELECT ALL THAT APPLY)***

1. In-person
2. Over the telephone
3. Virtually (Facetime, Zoom, etc.)
4. Other (SPECIFY)
5. (Refused)

Q13.
Do you know the cause of dementia for the person you care for? For example, was it dementia due to Alzheimer’s Disease?

1. (RECORD RESPONSE VERBATIM)
2. (Don’t Know)
3. (Refused)

Q14.
At about what age was the person you care for when dementia was diagnosed?

1. (ENTER AGE)
2. (Refused)

Q15.
How old is the person you care for now?

1. (ENTER AGE)
2. (Refused)

Q16.
How old are you?

1. (ENTER AGE)
2. (Refused)

Q17.
What is your biological sex?

1. Male
2. Female
3. Other
4. (Refused)

Q18.
Do you identify as man, woman, or as another gender?

1. Man
2. Woman
3. Another gender
4. (Refused)

Q19.
Have you ever used technology that you can wear? This could be things like an Apple watch, an iPod in a carrier, or other technology that you carry with you day-to-day.

1. Yes
2. No ***SKIP TO Q23***
3. (Refused) ***SKIP TO Q23***

Q20.
Could you tell me what you used it for?

1. (RECORD RESPONSE VERBATIM)
2. (Refused)

Q21.
How did you find the experience of using it?

1. (RECORD RESPONSE VERBATIM)
2. (Refused)

Q22.
On a scale of 1-10 where 1 is not comfortable at all and 10 is very comfortable, how comfortable are you using this wearable technology?

1. 1 – not comfortable
2. 2
3. 3
4. 4
5. 5
6. 6
7. 7
8. 8
9. 9
10. 10 – very comfortable
11. (Don’t Know)
12. (Refused)

Q23.
Have you ever used smart home technology? These include devices such as an Alexa, Google Home or iHome.

1. Yes
2. No ***SKIP TO Q27***
3. (Refused) ***SKIP TO Q27***

Q24.
Could you tell me what you used it for?

1. (RECORD RESPONSE VERBATIM)
2. (Refused)

Q25.
How did you find the experience of using it?

1. (RECORD RESPONSE VERBATIM)
2. (Refused)

Q26.
On a scale of 1-10 where 1 is not comfortable at all and 10 is very comfortable, how comfortable are you using this smart home technology?

1. 1 – not comfortable
2. 2
3. 3
4. 4
5. 5
6. 6
7. 7
8. 8
9. 9
10. 10 – very comfortable
11. (Don’t Know)
12. (Refused)

Q27.
Are there specific things you like about technology?

1. (RECORD RESPONSE VERBATIM)
2. (Refused)

Q28.
Are there specific things you dislike about technology?

1. (RECORD RESPONSE VERBATIM)
2. (Refused)

Q29.
Did you use technology more during the COVID-19 pandemic? Why or why not?

1. Yes (RECORD WHY)
2. No (RECORD WHY NOT)
3. (Refused)

Q30.
On a scale of 1-10 where 1 is not helpful at all and 10 is very helpful, how helpful do you think technology would be for your caregiving?

1. 1 – not helpful
2. 2
3. 3
4. 4
5. 5
6. 6
7. 7
8. 8
9. 9
10. 10 – very helpful
11. (Don’t Know)
12. (Refused)

Q31.
Do you have any fears or concerns about using a technology for caregiving?

(if yes) Could you describe them for me?

1. Yes (RECORD RESPONSE VERBATIM)
2. No
3. (Refused)

Q32.
Thank you for answering my questions about technology and dementia. Is there anything you would like to say about technology that I didn’t ask?

1. (RECORD RESPONSE VERBATIM)
2. (Refused)

I1.
Would you be willing to be contacted in the future for other studies relating to dementia?

1. Yes ***CONTINUE***
2. No ***SKIP TO END***
3. (Refused) ***SKIP TO END***

I2.
May I please have your name, phone number, and/or email address?

1. (Enter name)
2. (Enter phone number)
3. (Enter email)
4. (Refused)

END.

Those are all the questions that I have! On behalf of the University of Saskatchewan, thank you for your time. Your responses are greatly appreciated! Have a great day/evening!
