# Supplementary material for: Perspectives on Technology Use in the Context of Caregiving for Persons With Dementia: Qualitative Interview Study
Source: JMIR Form Res. 2024 Dec 13;8:e63041. doi: 10.2196/63041 (PMC11681293; doi:10.2196/63041)
Supplement: Multimedia Appendix 2 [file formative_v8i1e63041_app2.docx]

**Appendix 2: In-Depth Semi-Structured Interview Guide**

[go over consent form]

Background:

1. Could you tell me your age?

2. What is your sex and your gender?

3. What is your postal code?

4. How long have you been helping, looking after or caring for a person with dementia? How did it start? What is their relationship to you?

5. Can you tell me a bit about your current living arrangements with your loved one with dementia? Do you live together or apart?

6. If you live apart, how do you do your caregiving?

7. What support, if any, do you receive from other people?

Questions:

1. What are your experiences with using technology in your daily life?

For the rest of our discussion, when I say technology, I am going to be referring to things that are electronic, connected to the internet, and/or you can use for communication. It might be used for accessing information or helping you day to day, like setting up reminders or making lists electronically.

I’d like to focus a bit more on your experiences and thoughts with certain types of technology like smartphones, smartwatches, and smarthomes like an Alexa or an iHome:

2. Do you use a smartphone like an iPhone, Samsung phone, Google phone, or a phone that is like a little computer?

3. Do you use wearable technology like a smartwatch or fitbit, or some other piece of technology that you wear?

4. Do you use smart home technology like an Alexa, google home, iHome?

| If use technology  What sort of things do you use your wearable technology for?  -skip if doesn’t use  Could you tell me about your experience using wearable technology?  -Skip if doesn’t use  What sort of things do you use your smart home technology for?  Could you tell me about your experience using smart home technology?  -Skip if doesn’t use  Do you use technology in your caregiving?  -If yes: what things?  -If no: How come?  Are there any ways that technology has made caregiving easier for you?  Are there any ways that technology has made caregiving more challenging for you?  What do you do if something goes wrong with your technology?  Where do you go if you have questions about technology?  Pretend that your [smartphone/watch, etc] disappeared, and that technology was suddenly gone. Do you think that would affect your caregiving? In what way/why not? | If don’t use technology  Do you see a use for technology in your life?  How comfortable are you using technology? How comfortable would you be if you had to use technology?  What are some of the reasons that you don’t use technology more often?  Prompts: - Is cost an issue?  -Are security/privacy concerns for you?  -Do you feel you don’t know enough about technology?  -Do you find technology too distracting? |
| --- | --- |

5. On the flipside: pretend someone gave you a smartphone and a smart home device. Do you think that you would use it for your caregiving?

6. Can you think of anything that would help you want to try using technology for caregiving?

7. Let’s say you were interested in technology to use for caregiving; what features would you look for? Any you would avoid?

8. Are there things that might stop you from using or adopting technology for caregiving?

9. Do you think it would be doable for you to add technology into your caregiving routine?

-why?

-why not?

10. Did the COVID-19 pandemic change how you used technology?

-If so, how?

-If not, how come?

11. Is there anything you would like to say around technology use and caregiving that hasn’t already been discussed?

Thank you for participating in this interview. Your responses will really help me gain an understanding of technology and caregiving Saskatchewan and I am grateful for your time. Would you be interested in being contacted to participate in another study on integrating technology with caregiving?

Potential Prompts:

-How interesting

-Tell me more about that?

-Could you tell me what you mean when you say…

-Could you tell me about….

-General nonword encouragements for elaboration common to interviewing (e.g., mhm, mmm etc)
